# An open carbon–phenolic ablator for scientific exploration

**DOI:** 10.1038/s41598-023-40351-x

**Published:** 2023-08-12

**Authors:** Erik Poloni, Felix Grigat, Martin Eberhart, David Leiser, Quentin Sautière, Ranjith Ravichandran, Sara Delahaie, Christian Duernhofer, Igor Hoerner, Fabian Hufgard, Stefan Loehle

**Affiliations:** 1 High Enthalpy Flow Diagnostics Group, Institute of Space Systems, University of Stuttgart, 70569 Stuttgart, Germany; 2 Present Address: Centre for Advanced Structural Ceramics, Department of Materials, Imperial College London, London, SW7 2AZ UK; 3 Present Address: Vikram Sarabhai Space Center, Indian Space Research Organisation, Thiruvananthapuram, 695022 India

**Keywords:** Aerospace engineering, Characterization and analytical techniques, Design, synthesis and processing, Fluid dynamics

## Abstract

Space exploration missions rely on ablative heat shields for the thermal protection of spacecraft during atmospheric entry flights. While dedicated research is needed for future missions, the scientific community has limited access to ablative materials typically used in aerospace. In this paper, we report the development of the HEFDiG Ablation-Research Laboratory Experiment Material (HARLEM), a carbon–phenolic ablator designed to supply the need for ablative materials in laboratory experiments. HARLEM is manufactured using rayon-based carbon fiber preforms and a simplified processing route for phenolic impregnation. We characterized the thermal protection performance of HARLEM in arcjet experiments conducted in the plasma wind tunnel PWK1 of the Institute of Space Systems at the University of Stuttgart. We assessed the performance of the new material by measuring surface recession rate and temperature using photogrammetry and thermography setups during the experiments, respectively. Our results show that HARLEM’s thermal protection performance is comparable to legacy carbon–phenolic ablators that have been validated in different arcjet facilities or in-flight, as demonstrated by calculations of the effective heat of ablation and scanning electron microscopy of as-produced samples. In-house manufacturing of carbon–phenolic ablators enables the addition of embedded diagnostics to ablators, allowing for the acquisition of data on internal pressure and more sophisticated pyrolysis analysis techniques.

## Introduction

Spacecraft entering planetary atmospheres experience high aerothermal loads and require dedicated thermal protection systems^1,2^. At entry velocities greater than 11 km/s, ablative heat shields are typically used for thermal protection due to the extreme heat fluxes spacecraft have to withstand^3^. A thorough understanding of the mechanisms combined under the term *ablation* and of how they impact the performance of heat shields is key for optimizing thermal protection systems and reducing the risks involved in the most challenging space missions.

Carbon-phenolic ablators are the state-of-the-art ablative materials and have frequently been chosen for thermal protection systems of discovery missions^3^. They are able to dissipate large amounts of heat by ablation and, due to their high carbon content, by radiation re-emission^4,5^. To assess the performance of ablative materials and study their complex interaction with high enthalpy flows, researchers replicate atmospheric entry conditions in plasma wind tunnels. The data generated in these experiments is crucial to validate numerical models, which are then used to design and optimize heat shield materials. However, obtaining accurate material parameters during an experiment remains a challenge, especially when analyzing internal processes, requiring specialized diagnostic equipment. To improve the quality of research, there is a need for more fundamental material data and novel diagnostic techniques that can measure previously inaccessible parameters.

Diagnostic techniques often require both material production and instrument implementation, which can be challenging to replicate. Moreover, the processing methods used to manufacture conventional ablators are typically not disclosed in open literature, as they are often proprietary and owned by companies or government agencies. For instance, reports describing the development of the carbon–phenolic ablator PICA^6–8^ do not enable its reproduction, even if the method has been patented^9,10^. Other carbon–phenolic ablators, including ASTERM, AQ61, and ZURAM^11^, as well as other ablator variations such as AVCOAT, Cork P50, MA-25S, MonA, SLA-561, and ACUSIL are proprietary materials owned by various organizations, including the NASA Ames Research Center, the German Aerospace Center, Airbus SE, Amorim Cork Composites, Textron Inc., Peraton Inc., and Lockheed Martin Corp. As a result, the research community’s ability to study these materials is limited, which can hinder the development of advanced thermal protection systems for space exploration missions.

In this study undertaken at the High Enthalpy Flow Diagnostics Group (HEFDiG), we present the HEFDiG Ablation-Research Laboratory Experiment Material (HARLEM), a carbon–phenolic ablator manufactured from commercially-available materials using a processing route based on previous work^12^. The used materials and the full processing route are reported in detail, allowing for sample production with equipment typically found in chemistry and material laboratories. The route described here can be easily upscaled and adapted to tailor material parameters such as apparent density as a function of the relative amounts of the used components^12^. We tested the produced samples in the plasma wind tunnel PWK1, an arcjet facility in the Institute of Space Systems at the University of Stuttgart, using a suite of diagnostic methods to characterize HARLEM in extreme environments and validate its thermal protection performance for atmospheric entries^13^. The surface temperature of the samples was measured using a thermal imaging camera during the arcjet experiments, and recession data was acquired with a high-resolution photogrammetric setup developed in-house^14,15^. Based on these analyses, we compared the performance of HARLEM to that of commercial ablators tested in facilities dedicated to research in hypersonics^8,11,15–20^. Additionally, we used scanning electron microscopy to assess the microstructure of HARLEM and compare it to PICA and ASTERM based on microstructural data found in literature^21,22^. Our results show that HARLEM can be used as a platform to study ablation mechanisms of carbon–phenolic ablators. The reproducibility of the processing route and the validation of the manufactured samples support the use of HARLEM as an open material for further research.

## Results and discussion

### Modifications in the processing route

Carbon–phenolic ablators are composite materials that consist of a carbon fiber network infused with a porous aerogel-like phase of phenolic resin. Although their high carbon content is key to promote radiation re-emission, part of the absorbed heat is transferred through the material and heats up the spacecraft structure underneath^18^. Therefore, the main objective of an ablator is to maximize the dissipated heat while complying with system mass constraints^23^. To this end, the apparent density of carbon–phenolic ablators is typically less than $0.5\,\text {g}/\text {cm}^3$^8^, which is achieved by impregnating a mixture of phenolic resin, solvent, and polymeric additives into the voids of the carbon fiber network. The curing of the resin and removal of the solvent yield a porous phase, and the processing route used to manufacture HARLEM was specifically tailored to produce such characteristics^12^.

**Figure 1:**
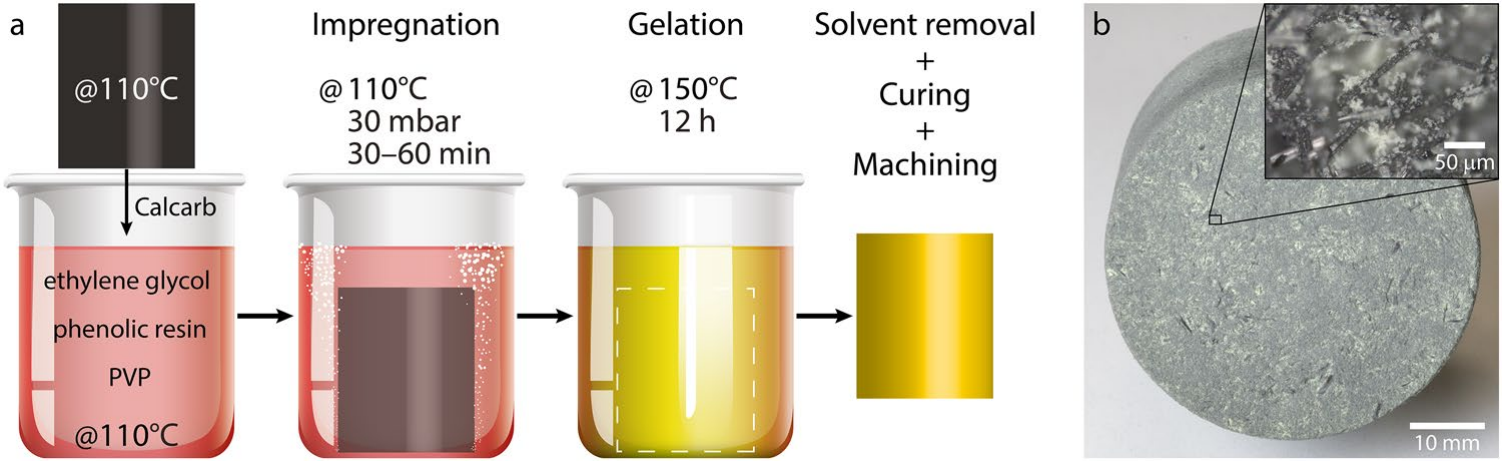
(**a**) Processing steps used to prepare near-net-shape samples of the HEFDiG Ablation-Research Laboratory Experiment Material (HARLEM). Adapted with permission from Poloni et al.^12^. (**b**) Frontal view of a HARLEM sample with density of $0.27\,\text {g}/\text {cm}^3$. Optical microscopy image featuring the resin-containing phase within the carbon fibers (inset).

With regards to the previously published processing route^12^, the modifications made in this work have allowed for a higher production output. Instead of dissolving phenolic resin, ethylene glycol, and poly(vinylpyrrolidone) (PVP) under vigorous stirring and initially heating them with the aid of a silicone oil bath, we simplified the process by directly placing the chemicals in a forced convection oven (see “Methods” section). Additionally, the rayon-based carbon fiber monolith from which preform specimens were taken was replaced with a carbon monolith of similar apparent density. However, we observed that replacing the preform yielded final ablators with a darker yellow color, likely due to the larger fiber diameters and to the fiber agglomerates reported for this monolith^24^. Based on these modifications in the processing route, we manufactured HARLEM samples by impregnating the preform specimens with a mixture of the chemicals used, gelating the resin phase in the presence of the solvent, and finally removing the solvent (Fig. 1a)^12^. All samples were produced with an apparent density of $0.27\,\text {g}/\text {cm}^3$, and the phase containing phenolic resin was well distributed within their volume, as confirmed by optical microscopy (Fig. 1b).

### HARLEM microstructure

**Figure 2:**
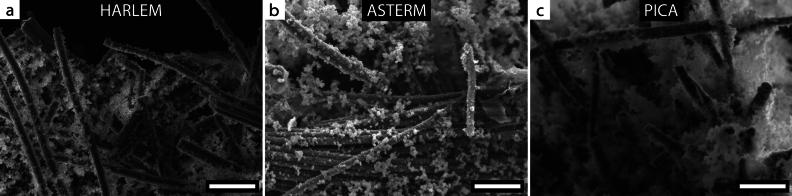
Electron microscopy images of the carbon–phenolic ablators (**a**) HARLEM, (**b**) ASTERM and (**c**) PICA. Adapted with permission from Agrawal et al.^21^ and Pinaud et al.^22^. Scale bars: $50\,\upmu \text {m}$.

The microstructure of HARLEM was found to be similar to that of other carbon–phenolic ablators. Electron microscopy images of the samples revealed that the resin-containing phase fills the spaces between the carbon fibers, a common feature in ASTERM^22^ and PICA^21^ samples (Fig. 2). The relatively good distribution of the phase containing phenolic resin in conventional carbon–phenolic ablators is responsible for promoting radiation scattering, which is known to reduce the total heat transfer through the ablator thickness^12^.

### HARLEM performance in arcjet experiments

During atmospheric entry, spacecraft must withstand extreme heat while keeping the substructure at a defined temperature level^20^. To meet this requirement without compromising the payload of spacecraft, carbon–phenolic ablators are designed to dissipate large amounts of heat with minimal weight addition^23^. As the ablative material is consumed, the material surface recedes^25^. On this basis, the effective heat of ablation is generally used in open literature to quantify the material performance during arcjet experiments^6,8,18^. The effective heat of ablation $h_{\text {eff}}$ is defined as:

1 $$\begin{aligned} h_{\text {eff}}=\frac{\dot{q}_{\text {cw}}}{\rho \dot{s}} \end{aligned}$$

where $\dot{q}_{\text {cw}}$ is the stagnation-point cold-wall heat flux, $\rho$ is the apparent density of the ablator and $\dot{s}$ is the surface recession rate for the given experimental conditions. Because of the high melting point and emittance of carbon, carbon–phenolic ablators are known to have the highest values of effective heat of ablation among ablative materials at heat fluxes above $4.5\,\text {MW}/\text {m}^2$, where a large portion of the energy is re-radiated. It has been found that, below this flux level, they are less efficient in terms of effective heat of ablation due to a more favorable oxidation and hence an increased recession rate^6^.

**Figure 3:**
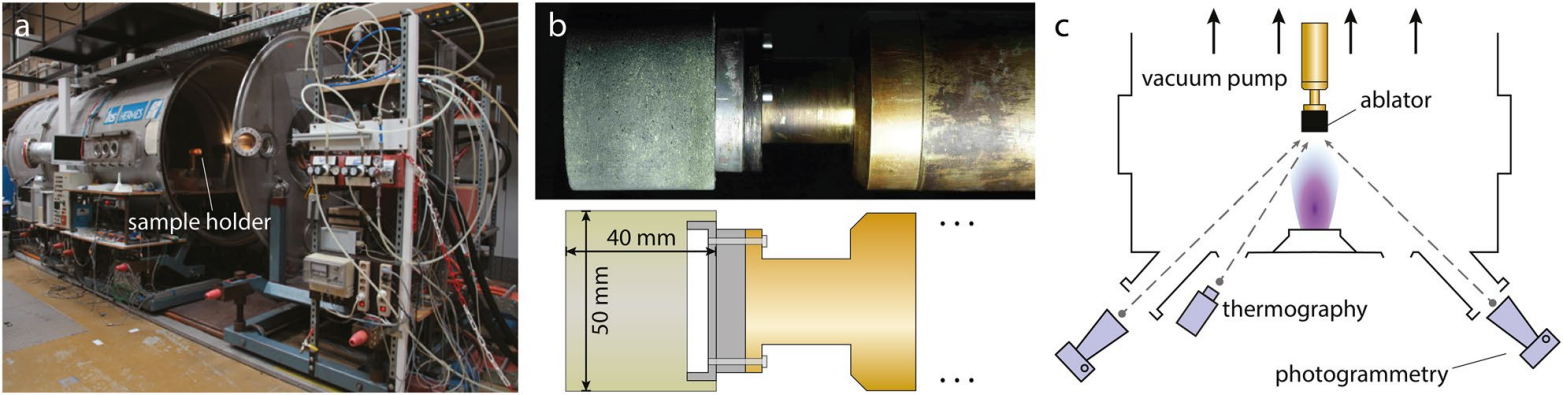
(**a**) Plasma wind tunnel facility PWK1. (**b**) Photograph and schematics of a HARLEM sample mounted on the sample holder. (**c**) Schematics of the instruments arrangement used for photogrammetry and thermography.

We evaluated the thermal protection performance of HARLEM by measuring its effective heat of ablation in experiments conducted in the plasma wind tunnel PWK1 at the Institute of Space Systems, University of Stuttgart. This facility houses a magnetoplasmadynamic arcjet generator mounted on the front lid of a vacuum chamber (Fig. 3a), which can generate flow enthalpies of up to 100 MJ/kg^13^. The flow conditions produced in plasma wind tunnels can be translated into stagnation-streamline flow fields of actual atmospheric entries using the Local Heat Transfer Simulation by Kolesnikov^26^. For the experiments in this study, we selected a flow condition that corresponds to a trajectory point of the Hayabusa capsule’s re-entry at an altitude of 78.8 km with a velocity of 11.7 km/s (Table 2)^27–29^. This flow condition has been extensively characterized in previous studies^30,31^ and generates a cold-wall heat flux of $5.4\,\text {MW}/\text {m}^2$. In addition to the heat flux, the average surface recession rate of HARLEM samples is also needed for the calculations in Eq. (1). Using high-resolution digital cameras, we measured the recession in-situ via photogrammetry^14^. To this end, two digital cameras were arranged such that a depth resolution of $53\,\upmu \text {m}$ was reached at the surface of the sample mounted on a dedicated holder (Fig. 3b,c)^15^. The average recession rate measured was $48\,\upmu \text {m}/\text {s}$. The stagnation-point cold-wall heat flux was obtained from the plasma wind tunnel experiments, and the apparent density of HARLEM was $0.27\,\text {g}/\text {cm}^3$. Using these values in Eq. (1), we calculated an effective heat of ablation of 417 MJ/kg for the tested HARLEM samples.

The effective heat of ablation is a critical parameter in evaluating the thermal protection performance of ablative materials. However, this property is not an inherent characteristic of materials alone and is influenced by the flow conditions under which they are tested in plasma wind tunnels. In order to better understand the performance of HARLEM, we compared its effective heat of ablation to that of other carbon–phenolic ablators along with the parameters used in Eq. (1), the total pressure ($p_{\text {tot}}$) and the mass-specific enthalpy (*h*) of the flow, the sample radius (*R*) and the maximum surface temperature ($T_{\text {s}}$) (Table 1). We considered the materials PICA^8,16,17^, developed by the NASA Ames Research Center, ASTERM^18–20^ and AQ61^19^, produced by Airbus SE, and ZURAM^11,15,20^, developed at the German Aerospace Center. Other ablators, such as AVCOAT^6,8,17^, Cork P50^32^, MA-25S^6^, MonA^17,33^, SLA-561^6^ and ACUSIL^6^, have been tested in arcjet experiments, but they do not belong to the class of carbon–phenolic ablators as they do not exclusively consist of a phenolic foam formed within a network of carbon fibers. For this reason, we excluded their performance parameters from the comparison. We calculated the surface temperature of the HARLEM samples from irradiance data obtained during the experiments using a thermography camera (Fig. 3c). We assumed an emissivity of 0.85 for all samples and a transmittance of 0.92 for the chamber windows. Our results show that HARLEM reached maximum surface temperatures ranging from 3200 to 3350 K. It is worth noting that the surface temperature not only depends on the material properties, such as the thermal conductivity, but also on the flow conditions determined by the energy balance at the ablator surface^6,8^.

**Table 1:** Thermal protection performance of carbon–phenolic ablators tested in air at plasma wind tunnel facilities.

Ablator	$\rho$ ($\text {g}/\text {cm}^3$)	$\dot{s}$ ($\upmu$m/s)	$\dot{q}_{\text {cw}}$ (MW/$\text {m}^2$)	$h_{\text {eff}}$ (MJ/kg)	$p_{\text {tot}}$ (hPa)	*h* (MJ/kg)	*R* (cm)	$T_{\text {s}}$ (K)
*HARLEM*	0.27	48	5.4	417	24.3	70	2.5	3200–3350
*ASTERM*^20^	0.35	32	5.2	464.3	24.3	70	2.5	2775
*ZURAM*^20^	0.38	43	5.2	318	24.3	70	2.5	3200
*ZURAM*^15^	0.4	25	5.4	540	24.3	70	2.5	2850–3000
*ZURAM*^11^	0.37–0.45	38–525	0.9–13.5	43.5–225.2	38–675	–	2.5	1847–3236
*PICA*^8^	0.244–0.371	120–380	4.3–33.6	43.9–382.7	111–436	–	1.27–5.08	2533–3311
*PICA*^16^	0.266	88–1040	4.0–16.3	58.9–254.3	203–659	29.5	1.27–5.08	1773–3023
*PICA*^17^	0.273	89	2.9	120	35	33.5	1.65	2488
*ASTERM*^18^	0.35	42	1.1–1.6	87	1013	16.4	2	2000–2100
*ASTERM*^19^	0.265	35–71	1.0–3.2	65.7–182.6	15–200	20.5–53.1	2.5	1845–2740
*AQ61*^19^	–	39–83	1.0–3.1	–	15–200	20.9–52.0	2.5	1860–2790

The reported parameters are apparent density ($\rho$), surface recession rate ($\dot{s}$), stagnation-point cold-wall heat flux ($\dot{q}_{\text {cw}}$), effective heat of ablation ($h_{\text {eff}}$), total pressure ($p_{\text {tot}}$), mass-specific enthalpy (*h*), sample radius (*R*) and maximum surface temperature ($T_{\text {s}}$).

The comparison of thermal protection performance shows that carbon-phenolic ablators demonstrate relatively high values of effective heat of ablation and maximum surface temperature when tested in high-enthalpy flows (Table 1). Specifically, the arcjet experiments conducted with HARLEM samples were performed with a mass-specific enthalpy of 70 MJ/kg, and at this condition its maximum surface temperature is slightly higher than that of ASTERM and ZURAM. While materials tested at different conditions cannot be quantitatively compared, most of the performance parameters of HARLEM fall within the range found for other ablators in the literature (Table 1).

**Figure 4:**
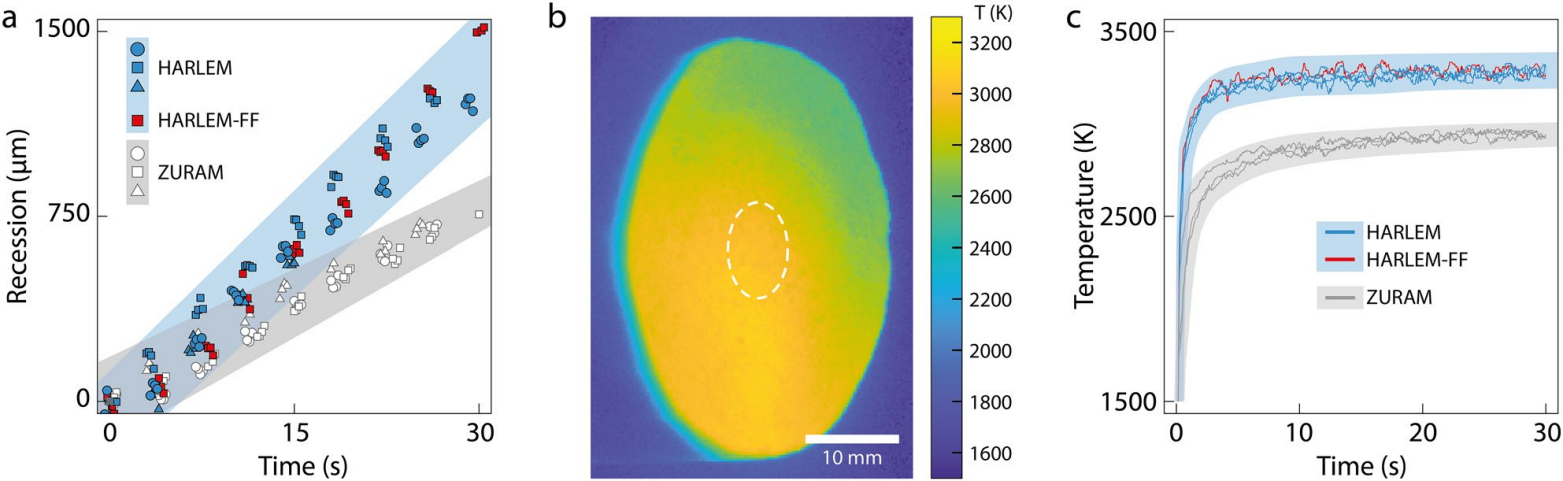
Performance of carbon–phenolic ablators tested at the plasma wind tunnel facility PWK1. (**a**) Recession evolution characterized using photogrammetry of standard HARLEM samples (blue), a HARLEM sample manufactured with the preform FiberForm^12^ (red) and ZURAM samples (gray). The recession data for ZURAM was taken from Grigat et al.^15^. (**b**) Temperature color map of a HARLEM sample acquired with an IR camera 5 s after the test start. The stagnation point is taken as a circular area in its center with a radius of 5 mm. (**c**) Stagnation-point temperature over time of the samples in (**a**) measured via thermography. The thermography data for ZURAM was taken from Grigat et al.^15^. The blue and gray shaded areas are guides to the eye and indicate trends for HARLEM and ZURAM, respectively.

The ablator ZURAM is one of the few materials that have already been tested under the same flow condition as those used for HARLEM in this study (Table 2)^15^. In the series of experiments including HARLEM and ZURAM, photogrammetry and thermography instruments were used to characterize the evolution of the surface recession rate and the surface temperature of the samples, respectively (Fig. 3c). Because both ablators are manufactured using the carbon preform Calcarb, they can be directly compared (Fig. 4). Recession measurements of HARLEM and ZURAM were plotted together and point out clear trends in surface recession (Fig. 4a). While HARLEM samples had an average recession rate of 48 μm/s, an average value of 25 μm/s has been measured for ZURAM samples^15^. Owing to the higher density of ZURAM, it has a lower surface recession rate and thus a higher effective heat of ablation (Table 1). Interestingly, a HARLEM sample manufactured using the rayon-based carbon preform FiberForm in the scope of a previous study^12^ displayed a recession behavior similar to that of typical HARLEM, indicating the reproducibility of our processing route for different preforms. This sample is referred to as HARLEM-FF (Fig. 4a,c).

**Table 2:** Test condition at PWK1 corresponding to the Hayabusa re-entry at 78.8 km.

Parameter	Value
Air mass flow ($\dot{m}$)	18.0 g/s
Ambient pressure ($p_{\infty }$)	16.6 hPa
Total pressure ($p_{\text {tot}}$)	24.3 hPa
Arc current (*I*)	1220 A
Arc voltage (*U*)	133 V
Electric power (*P*)	163 kW
Probe position	270 mm
Cold-wall heat flux on 50 mm ($\dot{q}_{\text {cw}}$)	5.4 MW/$\text {m}^2$
Mass-specific enthalpy (*h*)	70 MJ/kg

A similar comparison was made for the average surface temperature of HARLEM and ZURAM. The temperature data considered for this analysis related to the stagnation point of the samples, defined as a circular area in its center with a radius of 5 mm (Fig. 4b). The temperature evolution during the experiments was obtained by averaging the temperature values over the stagnation point for each frame (Fig. 4c). This analysis revealed a difference in the temperature curves obtained for the two ablators, which is a consequence of the higher apparent density of ZURAM. Because HARLEM and ZURAM are produced with the same carbon preform, a higher density implies the presence of more phenolic resin in the virgin ablator. The additional resin increases the connectivity between the front and the back of the samples^12^, leading to increased phonon transport and effective thermal conductivity of the samples. With more heat being transported towards the back of the ZURAM samples, their surface temperature, which is directly associated with the radiation re-emission, plateaued at 2850–3000 K, which is lower than the values of 3200–3350 K found for HARLEM (Fig. 4c).

## Conclusions

A simplified processing route was developed for the manufacture of carbon–phenolic ablators without the need of overpressure. Samples of the novel ablator HARLEM were produced and tested in the plasma wind tunnel PWK1 at the Institute of Space Systems, University of Stuttgart. Photogrammetry and thermography diagnostics were used to characterize the recession rate and surface temperature of HARLEM during arcjet experiments. The results demonstrated that HARLEM performs comparably to conventional carbon–phenolic ablators used by companies and government agencies. The microstructure of HARLEM was analyzed using optical and electron microscopy, which confirmed that the resin-containing phase is well-distributed in the material.

While this work showcases the principal capabilities of our carbon-phenolic ablator, a major motivation for the development of HARLEM is its use as a platform to gain insight into the complex phenomena of material ablation. We provide the complete processing route for manufacturing HARLEM, which will tremendously facilitate dedicated research on ablation. Following the published routine, this ablator is accessible to research laboratories worldwide. Future research based on HARLEM will be able to leverage the ability to tailor sample geometry, density, and chemistry to employ in-situ diagnostic techniques during arcjet and shock tube experiments. This approach will contribute to a deeper understanding of radiation-related processes, spectroscopy data, blowing parameters, pyrolysis outgassing, and other material-specific characteristics. We expect that these findings will help improve numerical ablation models and thereby impact the design of thermal protection systems.

## Methods

### Materials

Resole phenolic resin (Cellobond $\text {SC1008P}^{\textrm{TM}}$, Hexion), ethylene glycol (>98%, VWR chemicals) and poly(vinylpyrrolidone) (PVP, $\text {M}_{W}=10{,}000\,\text {g}/\text {mol}$, Sigma Aldrich) were used without further purification. Cylindrical preform specimens with diameter of 62 mm and height of 50 mm were taken from a carbon fiber porous monolith with an apparent density of $0.18\,\text {g}/\text {cm}^3$ (Calcarb${\circledR }$ CBCF 18-2000, Mersen) based on rayon. The specimens were punched directly from the carbon fiber monolith with the help of a thin-walled steel tube. The handling and manipulation of the materials were conducted in accordance with the guidelines provided in their respective Material Safety Data Sheets (MSDS).

### Manufacturing of samples

Samples of the carbon–phenolic ablator HARLEM were manufactured by adapting a previously described processing route^12^. The adaptations consisted in decreasing the number of processing steps, eliminating the need for stirring and silicon oil bath, and using commercially available carbon fiber monoliths.

To produce a typical HARLEM sample, two 400-mL glass beakers were first filled with 101.75 g of ethylene glycol each. 39.78 g of resole phenolic resin was added to one of the beakers and 3.98 g of PVP was added to the other. The beakers were covered with aluminum foil to avoid evaporation and were placed side by side, together with a preform specimen, in a forced convection oven (universal oven UF30, Memmert) at $150\,^{\circ }\text {C}$ under maximum ventilation. The resin and the PVP dissolved in ethylene glycol and a digital thermometer placed in one of the beakers indicated that the solutions reached $110\,^{\circ }\text {C}$ within 30 min inside the oven. After reaching $110\,^{\circ }\text {C}$, the content of one beaker was poured into the other and the preform specimen was immersed in the formed solution. Note that this step can lead to an irreversible phase separation and the creation of undesired precipitates if not conducted above $110\,^{\circ }\text {C}$. Next, the beaker containing the solution and the preform was covered with pierced aluminum foil and transferred to a vacuum drying oven (1450 W, Goldbrunn) set at $150\,^{\circ }\text {C}$, where it was kept for 60 min. The preform was impregnated with the solution by continuously decreasing the oven pressure to 100 mbar within 30 min and further to 30 mbar within the next 30 min. Pressures below 30 mbar should be avoided so that the vapor pressure of the solvent is not reached. After the impregnation step, the beaker was transferred back to the forced convection oven and kept at $150\,^{\circ }\text {C}$ for 24 h for the polymerization of the phenolic resin in the presence of ethylene glycol. Subsequently, the sample was taken from the beaker and the excess of polymerized resin around it was removed. This facilitated the evaporation of the ethylene glycol in the vacuum drying oven, which was conducted at $150\,^{\circ }\text {C}$ and a pressure lower than 10 mbar for 24 h. Finally, the HARLEM sample was machined to a final diameter and height of 50 and 40 mm, respectively (Fig. 3b).

Because the density and the porosity of the samples are determined by the relative ratios between the phenolic resin, ethylene glycol and PVP amounts used in the manufacturing^12^, different compositions of HARLEM can be produced. The approach described here is also scalable, as the addition of PVP allows for the curing of the phenolic resin to be performed without the need of overpressure. In this study, all HARLEM samples were manufactured with an apparent density of 0.27 g/$\text {cm}^3$^12^.

### Characterization of as-produced samples

The distribution of the resin phase within the volume of HARLEM samples was verified under an optical microscope (VHX-6000, Keyence) (Fig. 1b). The microstructure of the samples was characterized under a scanning electron microscope (SEM, LEO 1530 Gemini microscope) equipped with an in-lens detector. The SEM was operated at an acceleration voltage of 20 kV, an aperture size of $30\,\upmu \text {m}$ and a working distance of 8 mm (Fig. 2a).

### Arcjet facility

HARLEM samples were tested at the plasma wind tunnel facility PWK1 at the Institute of Space Systems of the University of Stuttgart (Fig. 3a)^13^. The facility consists in a plasma generator inside a vacuum chamber and is capable of producing flow conditions that duplicate the stagnation-streamline flow field of atmospheric entries using the Local Heat Transfer Simulation^26^. The plasma generator RD5, a magnetoplasmadynamic arcjet, is mounted to the front lid of the chamber and the electric power it needs is provided by an in-house current-regulated power supply^34^. The chamber has a diameter of 2 m, a length of 6 m and is connected to a vacuum pumping system. The characterization of samples during experiments is conducted via optical windows in the vacuum chamber^13^.

In a typical arcjet experiment in PWK1, the sample holders are screwed to a water-cooled probe that can be moved inside the tunnel to adjust for the desired heat load and total pressure condition on the flow axis (Fig. 3b). This configuration also allows for keeping the sample away from the plasma and thus unaffected by it during the startup procedure of the wind tunnel.

### Test condition

The parameters of the flow condition used in this study and the electric power settings to generate it are summarized in Table 2. This condition corresponds to a trajectory point of the Hayabusa capsule re-entry in 2010 at an altitude of 78.8 km with a velocity of 11.7 km/s^27–29^.

The flow condition was adjusted using a heat flux and total pressure gauge with an outer diameter of 80 mm. The stagnation-point cold-wall heat flux was measured with a water-cooled calorimeter inserted in it, while a bore in the center of the calorimeter allowed for the measurement of the total pressure. Because the HARLEM samples had a diameter of 50 mm, the actual stagnation-point cold-wall heat flux was scaled from the value measured with the 80-mm gauge using the relation $\dot{q}_{\text {cw}}\propto 1/\sqrt{R}$^35^.

### Characterization during arcjet experiments

The surface recession rate of the samples was characterized with a photogrammetry setup consisting of two high-resolution DSLR cameras (Canon EOS 5DSR, 50 MP) with optical access to the ablator surface through the windows in the lid of the chamber (Fig. 3c). The cameras were triggered in groups of 3 to 4 photos at a frequency of 5 Hz with an interval of $\sim$3.5 s between each group. After matching corresponding image points in the ablator images taken with the cameras, a three-dimensional point cloud of the ablator surface was reconstructed for each image set taken during the experiment^14,15^. This setup had a raw pixel resolution of ${12.5}\,\upmu \text {m}\,\text {px}^{-1}$, which corresponds to a total lateral resolution of ${75}\,\upmu \text {m}$ and a depth resolution of ${53}\,\upmu \text {m}$ after considering the optical aberration of the lenses^15^.

The surface temperature of the samples was acquired with a thermography camera (Mikron MCS640-HD, LumaSense Technologies) with a resolution of 640 × 480 px at frame rate of 60 fps (Fig. 3c). An emissivity of 0.85 was taken for all samples and a transmittance of 0.92 was taken for the chamber windows.

### Experimental sequence

In each experiment, the sample was mounted on the probe and positioned next to the wall of the chamber such that it was kept away from the plasma before ignition. Next, the lid was closed, the chamber was evacuated to approximately 0.1 hPa, the plasma was ignited and the pressure, air mass flow and power settings were adjusted to the desired levels (Table 2). Finally, the data acquisition using the diagnostic equipment was started and the sample was moved to its final position to face the plasma flow, which marked the start of the test. After a test duration of 30 s, the plasma generator was shut off, the gas flow was shut off and the sample was left to cool for 5 min before the facility was vented.

## Supplementary Information

Supplementary Tables.

## Data Availability

Data supporting the results reported in the article can be found in the Supporting Information.
