# Correction: An open carbon–phenolic ablator for scientific exploration

**DOI:** 10.1038/s41598-026-61513-7

**Published:** 2026-07-20

**Authors:** Erik Poloni, Felix Grigat, Martin Eberhart, David Leiser, Quentin Sautière, Ranjith Ravichandran, Sara Delahaie, Christian Duernhofer, Igor Hoerner, Fabian Hufgard, Stefan Loehle

**Affiliations:** 1 High Enthalpy Flow Diagnostics Group, Institute of Space Systems, University of Stuttgart, 70569 Stuttgart, Germany; 2 Present Address: Centre for Advanced Structural Ceramics, Department ofMaterials, Imperial College London, London, SW7 2AZ UK; 3 Present Address: Vikram Sarabhai Space Center, Indian Space Research Organisation, Thiruvananthapuram, 695022 India

Correction to: *Scientific Reports* 10.1038/s41598-023-40351-x, published online 12 August 2023

The original version of this Article contained a repeated nomenclature error where the Calcarb CBCF 18-2000 preform (Mersen) was incorrectly identified as being based on polyacrylonitrile (PAN).

As a result, in the Abstract,

“HARLEM is manufactured using polyacrylonitrile-based carbon fiber preforms and a simplified processing route for phenolic impregnation.”

now reads:

“HARLEM is manufactured using rayon-based carbon fiber preforms and a simplified processing route for phenolic impregnation.”

Furthermore, in the Results and Discussion, under the subheading ‘Modifications in the processing route’,

“Additionally, the rayon-based carbon fiber monolith from which preform specimens were taken was replaced with a carbon monolith based on polyacrylonitrile (PAN) fibers. This replacement did not require any special modifications, as both carbon monoliths have a similar apparent density.”

now reads:

“Additionally, the rayon-based carbon fiber monolith from which preform specimens were taken was replaced with a carbon monolith of similar apparent density.”

In addition,

“However, we observed that the use of a PAN-based monolith yielded final ablators with a darker yellow color, likely due to the larger fiber diameters and to the fiber agglomerates reported for this monolith^24^.”

now reads:

“However, we observed that replacing the preform yielded final ablators with a darker yellow color, likely due to the larger fiber diameters and to the fiber agglomerates reported for this monolith^24^.”

and the following sentence has been deleted:

“By replacing the source of carbon fibers, we also ensured that the used monolith was commercially available, which was not the case in the previous route due to international regulations.”

Finally, under the subheading ‘HARLEM performance in arcjet experiments’,

“Because both ablators are manufactured using the PAN-based carbon preform Calcarb, they can be directly compared (Fig. 4).”

now reads:

“Because both ablators are manufactured using the carbon preform Calcarb, they can be directly compared (Fig. 4).”

In the Methods, under the subheading ‘Materials’,

“Cylindrical preform specimens with diameter of 62 mm and height of 50 mm were taken from a carbon fiber porous monolith with an apparent density of 0.18 g/cm3 (Calcarb® CBCF 18-2000, Mersen) based on polyacrylonitrile (PAN).”

now reads:

“Cylindrical preform specimens with diameter of 62 mm and height of 50 mm were taken from a carbon fiber porous monolith with an apparent density of 0.18 g/cm3 (Calcarb® CBCF 18-2000, Mersen) based on rayon.”

Lastly, under the subheading ‘Manufacturing of samples’,

“The adaptations consisted in decreasing the number of processing steps, eliminating the need for stirring and silicon oil bath, and using commercially available carbon fiber monoliths based on PAN instead of rayon fibers.”

now reads:

“The adaptations consisted in decreasing the number of processing steps, eliminating the need for stirring and silicon oil bath, and using commercially available carbon fiber monoliths.”

The original Article has been corrected.

