# Supplementary material for: An open carbon–phenolic ablator for scientific exploration
Source: Sci Rep. 2023 Aug 12;13:13135. doi: 10.1038/s41598-023-40351-x (PMC10423272; doi:10.1038/s41598-023-40351-x)
Supplement: Supplementary file 1 — Supplementary Tables. [file 41598_2023_40351_MOESM1_ESM.pdf]

An open carbon–phenolic ablator for scientific exploration

Erik Poloni[1,2,*], Felix Grigat[1], Martin Eberhart[1], David Leiser[1], Quentin Sautière[1], Ranjith Ravichandran[1,3], Sara Delahaie[1], Christian Duernhofer[1], Igor Hoerner[1], Fabian Hufgard[1], and Stefan Loehle[1]

[1]High Enthalpy Flow Diagnostics Group, Institute of Space Systems, University of Stuttgart, 70569, Stuttgart, Germany
[2]Now at: Centre for Advanced Structural Ceramics, Department of Materials, Imperial College London, SW7 2AZ, London, United Kingdom
[3]Now at: Vikram Sarabhai Space Center, Indian Space Research Organisation, 695022, Thiruvananthapuram, India
*e.poloni@imperial.ac.uk

The temperature and recession data acquired for the different HARLEM samples are shown in Tables S1 and S2, respectively.

Table S1. Temperature data acquired for HARLEM samples.

Temperature (K)			
**HARLEM 1**	**HARLEM 2**	**HARLEM 3**	**HARLEM 4**
1470.23	1469.11	1467.72	1464.81
1471.89	1464.73	1466.40	1459.72
1457.69	1452.23	1448.22	1445.61
1471.38	1466.86	1458.13	1460.53
1472.13	1467.92	1461.15	1457.64
1478.40	1469.40	1470.18	1466.66
1471.75	1470.76	1483.72	1473.64
1470.68	1462.44	1510.43	1468.61
1466.18	1461.68	1884.88	1463.41
1479.76	1464.29	2297.67	1453.69
1465.55	1460.48	2341.16	1479.83
1463.67	1455.46	2436.39	1611.52
1467.64	1464.22	2478.22	2055.07
1469.63	1466.88	2560.88	2342.67
1471.56	1460.41	2615.42	2478.91
1473.54	1445.13	2686.89	2472.33
1475.88	1459.66	2640.37	2547.84
1472.49	1468.51	2742.01	2615.49
1462.51	1463.42	2647.97	2704.45
1471.86	1478.04	2707.68	2662.73
1467.91	1472.40	2807.96	2798.68
1475.74	1469.09	2822.26	2603.97

1468.76	1537.10	2892.27	2744.51
1468.71	1795.51	2988.51	2760.77
1499.64	2135.04	2858.46	2854.08
1595.62	2204.42	2879.96	2856.83
2109.56	2418.96	2888.54	2739.72
2448.96	2340.93	2831.72	2836.00
2331.63	2473.38	2902.65	2837.25
2523.80	2468.50	2943.11	2846.08
2442.95	2488.87	2939.95	2891.16
2562.06	2562.39	3021.70	2724.53
2600.78	2537.96	2931.93	2803.86
2666.84	2604.40	3029.74	2817.76
2643.13	2824.86	3052.39	2788.09
2786.30	2756.05	3052.16	2936.43
2857.70	2812.73	2967.19	2918.25
2967.19	2831.83	3008.92	2953.15
2878.89	2833.78	2993.88	2812.80
2899.28	2790.86	3064.10	2889.38
2844.21	2928.11	2974.69	2923.08
2892.02	2957.00	2991.95	2999.60
2898.89	2932.33	3086.67	2926.25
2968.42	2892.77	3064.37	2992.91
2990.99	2915.62	2985.82	2882.66
2946.34	2872.37	3021.79	2956.64
2904.27	2915.93	2993.02	2995.71
2872.46	2928.04	3148.15	2908.22
3051.15	2963.39	3076.32	3007.17
2773.45	3003.30	3003.96	3050.78
3038.48	2946.31	3124.83	3084.93
2928.84	2954.09	3046.70	2954.89
2930.53	2967.23	3181.81	3007.14
3009.35	3004.91	2932.89	3057.94
2991.57	2991.45	3107.16	3018.05
3046.66	3023.84	3038.73	2980.35
3095.70	3052.11	2976.54	3063.19
3082.00	3010.64	3049.70	2981.06
3087.60	3122.26	3077.67	3000.84
3132.30	3115.10	3090.59	3108.80
3142.31	2967.55	3020.98	3001.55
3017.77	3093.94	3225.41	3109.99
3031.72	2990.88	3149.54	3132.30
3136.36	2993.84	3128.22	3074.44
3033.15	3086.70	3178.24	3096.45
3055.67	3032.91	3056.89	3174.80
3025.85	3040.49	3138.72	3048.93
3028.77	3028.14	3164.62	3098.67
3031.61	2988.47	3218.01	3148.51

3187.65	3071.19	3137.85	3134.74
3205.93	3136.86	3137.23	3150.81
3082.45	2987.44	3054.54	3152.09
2990.77	3035.46	3045.72	3143.11
3154.21	3050.06	3168.99	3165.17
3043.75	3030.35	3131.92	3011.12
3118.71	3082.14	3270.36	3097.30
3099.90	3122.48	3152.52	3143.28
3195.95	3061.46	3182.27	3177.95
3085.59	3127.51	3070.20	3196.62
3108.81	3109.61	3157.80	3060.87
3123.20	3121.57	3171.91	3082.81
3142.29	3073.48	3116.84	3097.43
3074.02	3241.97	3326.65	3062.99
3193.35	3094.86	3120.40	3156.55
3249.93	3197.42	3098.85	3093.04
3104.28	3146.56	3197.87	3094.34
3049.90	3161.68	3236.74	3149.01
3235.33	3132.66	3220.06	3112.17
3202.75	3072.97	3084.52	3229.62
3156.93	3111.86	3102.49	3077.37
3269.04	3174.24	3066.86	3213.53
3148.81	3039.28	3232.15	3114.51
3179.77	3055.77	3259.27	3130.12
3265.60	3148.72	3248.53	3237.84
3087.66	3041.95	3198.38	3126.65
3121.11	3168.15	3128.82	3121.53
3192.76	3178.68	3148.01	3026.71
3065.44	3097.08	3127.78	3118.15
3202.67	3128.32	3087.88	3133.23
3127.56	3094.20	3176.63	3285.85
3138.09	3166.57	3276.90	3122.35
3165.15	3176.57	3209.46	3216.45
3094.58	3063.79	3133.07	3162.95
3030.53	3084.23	3132.84	3177.21
3240.15	3061.18	3011.06	3093.90
3249.40	3141.33	3276.22	3001.50
3263.20	3184.55	3261.32	3143.74
3274.05	3151.20	3230.26	3232.90
3239.83	3139.06	3171.73	3181.49
3255.56	3038.10	3195.74	3147.57
3237.99	3089.57	3311.86	3289.29
3246.81	3256.49	3174.18	2981.09
3220.76	3200.35	3233.56	3153.24
3160.45	3179.39	3198.16	3164.29
3208.69	3115.63	3233.18	3090.75
3276.01	3095.42	3274.45	3039.31

3267.39	3194.51	3261.03	3185.55
3227.71	3239.72	3178.00	3285.02
3077.71	3227.92	3205.01	3149.77
3227.26	3240.05	3242.68	3153.10
3067.84	3242.21	3168.49	3189.03
3310.45	3146.56	3038.61	3174.76
3084.24	3117.84	3000.61	3215.72
3229.07	3032.32	3101.44	3196.62
3293.36	3247.10	3076.60	3150.90
3147.27	3183.67	3259.42	3285.78
3293.35	3343.56	3093.20	3170.17
3300.03	3194.69	3240.37	3307.74
3246.39	3131.38	3160.94	3072.93
3149.06	3263.20	3205.53	3020.29
3064.66	3238.37	3281.75	3070.64
3245.83	3233.03	3230.48	3126.58
3282.69	3231.80	3283.58	3196.27
3303.16	3268.39	3167.77	3095.42
3223.67	3234.55	3177.08	3007.49
3199.07	3250.92	3081.60	3152.72
3113.03	3102.30	3279.17	3344.83
3104.23	3215.37	3244.72	3155.20
3191.18	3112.95	3167.78	3180.67
3074.54	3140.39	3276.83	3267.21
3242.81	3181.69	3266.55	3238.31
3183.44	3140.49	3165.16	3283.76
3107.83	3175.69	3265.76	3155.60
3242.11	3066.95	3242.51	3153.22
3093.17	3158.10	3261.87	3291.38
3098.22	3350.56	3090.17	3340.16
3221.64	3192.59	3195.80	3164.51
3244.91	3189.98	3130.94	3268.35
3028.14	3123.46	3206.49	3177.27
2976.10	3257.62	3185.16	3036.51
3077.79	3024.62	3178.44	3139.41
3130.17	3160.73	3258.75	3138.56
3247.11	3233.45	3194.79	3197.80
3020.74	3177.14	3279.07	3162.56
3226.85	3061.53	3256.67	3281.19
3207.72	3132.66	3137.78	3143.70
3203.97	3206.59	3217.88	3014.60
3305.51	3215.51	3124.55	3281.87
3265.31	3295.90	3223.08	3174.29
3112.81	3110.96	3231.38	3163.65
3233.71	3181.29	3122.74	3177.82
3233.71	3203.19	3295.31	3106.15
3286.19	3261.34	3239.78	3350.06

3307.30	3071.66	3333.25	3126.13
3335.65	3187.11	3272.48	3177.17
3294.30	3046.07	3251.21	3113.94
3162.57	3223.77	3197.74	3159.03
3335.13	3203.17	3243.03	3101.44
3055.16	3321.41	3165.56	3280.81
3155.54	3188.46	3089.19	3336.39
3127.48	3171.90	3110.17	3250.13
3258.01	3265.58	3079.20	3202.37
3371.77	3145.64	3265.29	3191.47
3189.57	3217.62	3219.14	3301.27
3313.37	3341.18	3130.56	3201.80
3290.05	3145.95	3223.58	3101.58
3265.38	3334.05	3287.45	3230.17
3271.34	3240.59	3266.31	3213.14
3294.14	3132.37	3207.13	3174.63
3285.29	3235.99	3190.81	3255.20
3125.97	3204.32	3295.33	3140.89
3241.43	3293.85	3254.05	3139.70
3083.75	3223.31	3150.53	3151.09
3108.11	3248.91	3175.63	3077.25
3170.68	3215.23	3264.19	3308.87
3198.56	3217.94	3215.78	3272.64
3240.76	3165.30	3229.18	3206.31
3154.66	3188.48	3290.04	3210.55
3167.85	3170.68	3163.12	3172.94
3242.21	3312.20	3193.04	3199.89
3253.64	3297.75	3258.82	3138.72
3265.96	3186.21	3332.83	3188.89
3178.59	3310.65	3180.87	3221.61
3250.86	3192.38	3193.06	3294.08
3346.96	3214.30	3329.32	3189.26
3217.37	3242.77	3281.53	3221.78
3153.92	3276.46	3258.62	3260.08
3307.67	3294.58	3256.26	3044.63
3339.69	3235.98	3265.37	3139.50
3274.20	3219.10	3108.17	3240.64
3205.74	3280.43	3201.34	3134.97
3263.50	3273.68	3205.01	3077.46
2967.32	3224.07	3276.34	3292.41
3175.68	3176.75	3346.69	3125.95
3250.52	3164.59	3267.44	3215.16
3302.12	3227.48	3280.29	3330.66
3079.47	3170.05	3146.32	3073.92
3237.73	3077.25	3280.04	3191.75
3312.71	3284.02	3221.57	3212.23
3236.13	3103.19	3285.90	3161.20

3063.12	3190.66	3260.82	3299.47
3323.31	3261.16	3274.98	3216.04
3350.58	3233.20	3275.46	3264.84
3175.29	3217.97	3294.99	3202.90
3298.91	3183.05	3214.81	3136.67
3265.28	3229.77	3297.04	3259.31
3311.14	3161.06	3267.08	3271.11
3239.18	3155.77	3280.20	3232.36
3177.46	3167.24	3320.23	3255.62
3174.85	3206.30	3252.04	3085.67
3197.69	3178.61	3406.21	3299.74
3273.79	3214.50	3224.33	3285.34
3188.92	3077.60	3171.80	3189.93
3265.27	3197.66	3331.29	3279.44
3289.03	3342.96	3180.93	3174.78
3249.47	3227.46	3112.91	3235.76
3243.89	3089.43	3381.30	3244.87
3328.94	3330.25	3269.10	3348.58
3208.98	3253.85	3185.31	3212.29
3252.90	3292.27	3173.06	3263.56
3250.51	3285.92	3139.63	3099.75
3350.47	3170.75	3124.16	3166.67
3021.68	3044.04	3256.31	3263.15
3280.97	3082.78	3297.68	3098.62
3106.97	3358.94	3299.14	3228.36
3216.50	3033.82	3115.69	3227.11
3141.13	3078.57	3362.25	3113.58
3247.68	3179.06	3174.71	3183.10
3296.89	3226.24	3038.53	3231.24
3186.11	3196.74	3229.73	3300.09
3184.30	3165.89	3160.88	3240.50
3253.89	3190.63	3310.39	3205.09
3253.75	3095.45	3330.06	3304.24
3293.48	3277.01	3167.90	3139.20
3349.12	3039.17	3200.04	3101.74
3327.43	3188.08	3257.61	3145.28
3171.46	3310.18	3301.42	3286.01
3308.00	3329.46	3251.88	3234.94
3432.70	3203.39	3281.32	3242.29
3185.92	3156.59	3196.63	3250.66
3364.60	3174.94	3178.50	3300.26
3106.19	3147.42	3314.61	3248.50
3388.01	3139.21	3325.79	3219.74
3188.84	3036.40	3208.90	3259.63
3297.13	3209.17	3309.66	3249.18
3305.38	3180.25	3269.15	3070.72
3317.02	3182.87	3288.06	3164.67

3276.43	3130.35	3167.37	3238.06
3258.33	3264.78	3070.89	3245.12
3342.03	3201.29	3221.47	3122.42
3208.31	3307.70	3338.14	3238.37
3344.20	3297.36	3376.96	3205.12
3321.55	3200.83	3146.71	3254.06
3216.37	3356.62	3230.72	3288.98
3253.25	3216.05	3167.20	3252.26
3254.92	3274.11	3173.08	3238.34
3320.34	3222.77	3199.06	3342.14
3243.82	3391.25	3225.27	3048.35
3202.89	3270.46	3221.57	3338.37
3172.92	3177.51	3216.62	3225.54
3262.16	3231.99	3226.31	3266.73
3060.92	3326.59	3151.37	3297.26
3323.39	3245.79	3216.13	3138.27
3164.19	3211.02	3174.85	3217.40
3223.43	3363.21	3096.92	3125.01
3200.99	3251.70	3286.43	3296.16
3210.77	3214.21	3177.44	3299.27
3295.54	3272.29	3255.28	3282.58
3233.66	3302.87	3423.13	3286.33
3191.35	3260.03	3267.64	3324.50
3254.19	3258.17	3233.72	3238.69
3268.74	3172.21	3416.62	3329.99
3147.79	3260.32	3157.90	3344.44
3151.60	3332.29	3129.45	3219.51
3347.09	3233.22	3289.67	3257.53
3109.81	3322.96	3236.70	3199.14
3164.08	3278.05	3291.10	3114.04
3310.15	3361.95	3349.00	3212.28
3269.64	3356.02	3335.54	3195.12
3225.18	3274.15	3312.85	3206.08
3261.98	3243.76	3181.49	3290.48
3332.19	3283.44	3275.68	3341.76
3231.50	3166.69	3278.36	3145.00
3238.84	3331.59	3117.88	3272.45
3377.20	3288.17	3179.61	3221.49
3139.13	3410.41	3230.64	3164.66
3241.43	3196.24	3087.76	3217.47
3375.46	3260.10	3368.11	3271.01
3275.38	3265.92	3130.67	3287.63
3187.00	3253.27	3214.92	3177.63
3180.39	3380.10	3122.38	3329.94
3267.63	3271.95	3311.42	3248.45
3335.12	3249.04	3086.89	2996.29
3286.70	3260.39	3258.07	3179.50

3139.30	3318.90	3264.94	3132.42
3080.78	3265.44	3056.94	3154.25
3216.21	3274.44	3277.83	3186.82
3230.17	3275.67	3203.23	3277.52
3211.74	3297.30	3289.43	3154.65
3180.24	3177.25	3212.49	3217.50
3340.19	3280.48	3277.23	3316.65
3262.53	3328.08	3334.47	3205.74
3141.43	3233.26	3193.94	3077.08
3297.05	3096.77	3256.91	3263.65
3265.22	3259.58	3180.74	3220.24
3246.11	3371.65	3184.67	3158.39
3054.42	3265.80	3290.69	3213.73
3287.30	3295.17	3322.74	3337.24
3227.22	3288.23	3228.91	3372.09
3216.88	3330.25	3300.80	3121.60
3177.31	3359.52	3273.24	3087.18
3235.50	3244.62	3220.29	3173.58
3404.15	3137.87	3293.05	3176.55
3412.70	3324.25	3334.60	3113.38
3155.50	3380.04	3319.95	3291.15
3360.52	3195.91	3250.03	3390.07
3272.77	3213.49	3165.00	3312.21
3198.81	3231.24	3220.08	3016.63
3328.66	3274.04	3184.03	3234.40
3180.92	3352.04	3231.26	3114.27
3219.67	3174.12	3178.24	3335.05
3334.12	3232.56	3223.56	3359.23
3365.07	3244.76	3353.49	3238.94
3313.44	3202.44	3260.43	3142.60
3269.59	3358.62	3219.24	3229.76
3396.22	3235.20	3206.89	3182.62
3370.39	3274.32	3266.56	3220.29
3346.22	3138.94	3436.82	3251.88
3241.04	3257.39	3216.77	3378.24
3377.62	3158.31	3124.23	3247.47
3329.17	3266.78	3335.32	3185.61
3193.45	3185.42	3211.21	3178.62
3288.57	3359.16	3265.99	3388.71
3291.81	3255.80	3255.58	3156.29
3218.69	3300.84	3228.55	3237.19
3094.31	3217.89	3118.06	3302.13
3188.61	3350.28	3397.47	3193.11
3258.43	3258.83	3353.11	3233.05
3336.47	3281.29	3295.45	3233.55
3147.82	3185.04	3289.60	3179.24
3234.25	3291.73	3365.48	3340.26

3158.54	3201.26	3272.10	3153.78
3342.69	3187.46	3310.73	3129.90
3240.23	3145.83	3123.75	3116.81
3257.53	3280.71	3160.48	3229.25
3269.33	3238.91	3353.62	3369.84
3382.86	3228.69	3198.21	3151.45
3291.71	3332.72	3247.33	3219.60
3180.62	3244.78	3310.33	3115.61
3351.61	3259.22	3326.80	3073.42
3251.41	3367.95	3329.67	3272.87
3244.18	3134.00	3318.69	3259.40
3334.07	3380.21	3333.11	3233.00
3252.02	3243.34	3303.84	3037.46
3274.99	3247.07	3245.05	3333.53
3317.16	3264.95	3357.84	3320.96
3179.34	3188.23	3316.80	3151.35
3149.91	3377.21	3090.07	3238.72
3216.22	3279.70	3292.84	3062.46
3417.03	3320.71	3322.63	3159.66
3313.22	3137.02	3208.07	3280.70
3186.34	3211.63	3204.05	3306.90
3285.84	3109.75	3275.08	3282.41
3131.55	3366.77	3326.61	3120.30
3330.78	3186.88	3253.33	3182.94
3227.12	3320.77	3318.33	3225.94
3351.56	3265.22	3133.99	3323.45
3331.06	3278.34	3264.41	3162.51
3167.85	3263.23	3184.70	3098.90
3302.62	3158.53	3372.33	3141.99
3206.43	3230.87	3341.65	3125.29
3249.90	3162.59	3271.79	3222.46
3248.74	3113.00	3247.72	3279.63
3306.26	3196.83	3304.98	3137.85
3281.76	3124.92	3251.21	3149.97
3117.73	3299.75	3222.30	3335.51
3435.03	3340.24	3297.28	3130.54
3109.24	3392.48	3311.75	3140.61
3351.87	3289.86	3244.10	3202.21
3381.10	3093.73	3274.37	3257.77
3284.29	3238.61	3206.01	3337.72
3311.68	3199.95	3368.38	3097.00
3119.18	3234.02	3187.67	3285.10
3302.76	3228.15	3255.16	3380.43
3262.73	3168.03	3405.75	3207.35
3317.97	3268.47	3252.28	3275.50
3160.00	3234.85	3240.26	3270.79
3263.72	3367.22	3427.64	3215.15

3248.83	3285.69	3399.96	3253.04
3272.88	3195.17	3353.71	3264.53
3332.24	3149.61	3358.62	3302.91
3194.23	3091.34	3310.98	3330.44
3302.62	3310.22	3096.10	3235.62
3229.94	3333.72	3278.53	3311.79
3363.56	3259.47	3305.18	3267.31
3327.56	3253.15	3306.75	3123.16
3322.93	3302.63	3280.53	3237.48
3332.89	3189.72	3213.59	3110.35
3230.61	3317.88	3459.37	3222.98
3338.12	3140.61	3320.05	3173.88
3307.61	3210.14	3374.20	3257.62
3232.29	3344.76	3429.26	3260.07
3212.86	3185.13	3476.45	3301.12
3058.43	3215.47	3296.58	3215.70
3285.36	3287.51	3230.08	3240.69
3346.89	3163.22	3159.57	3264.13
3391.88	3240.38	3368.52	3238.31
3337.94	3269.78	3279.42	3302.22
3374.27	3287.85	3335.49	3186.55
3344.03	3256.13	3284.19	3163.77
3186.96	3325.30	3248.76	3275.80
3338.89	3219.10	3212.89	3045.67
3340.93	3301.94	3260.17	3175.03
3354.64	3213.01	3277.81	3234.08
3216.60	3297.34	3181.86	3147.01
3208.45	3134.22	3276.27	3110.67
3325.28	3144.33	3113.13	3133.31
3370.07	3243.79	3306.46	3296.04
3253.71	3270.13	3315.33	3073.81
3384.07	3288.02	3305.01	3220.06
3321.27	3263.62	3103.96	3291.87
3343.06	3235.92	3251.94	3152.69
3227.51	3237.68	3110.46	3200.73
3171.02	3367.77	3257.07	3353.15
3140.86	3184.29	3145.12	3225.93
3208.22	3234.08	3344.41	3188.83
3129.77	3264.61	3341.29	3233.21
3288.98	3164.66	3227.44	3179.47
3087.42	3372.68	3198.06	3152.81
3377.64	3367.88	3209.52	3181.17
3277.99	3224.58	3307.97	3172.53
3191.71	3190.39	3353.51	3120.81
3255.79	3377.13	3366.06	3209.66
3235.20	3343.98	3285.68	3278.37
3382.55	3340.27	3149.70	3256.42

3140.64	3127.42	3264.92	3194.86
3319.62	3140.63	3166.80	3282.14
3283.98	3262.34	3272.22	3216.61
3274.93	3186.42	3203.63	3304.00
3035.63	3115.29	3139.96	3124.41
3291.87	3282.61	3218.20	3249.48
3271.22	3298.11	3198.45	3277.93
3233.22	3206.94	3155.09	3332.97
3311.71	3142.99	3338.62	3277.71
3268.51	3213.05	3234.05	3220.60
3302.57	3244.64	3235.17	3328.29
3344.66	3326.90	3215.28	3388.20
3262.14	3162.34	3306.83	3236.69
3323.16	3248.19	3346.56	3144.78
3319.32	3257.90	3162.57	3323.96
3214.27	3359.91	3251.06	3297.13
3236.67	3204.34	3318.41	3322.55
3192.91	3281.60	3394.49	3309.79
3328.79	3137.33	3293.40	3162.27
3294.79	3221.90	3219.19	3179.44
3351.76	3271.82	3222.66	3253.94
3210.34	3362.46	3130.02	3422.56
3394.97	3317.62	3248.32	3258.41
3237.19	3269.18	3209.90	3273.41
3268.83	3265.58	3135.34	3095.59
3368.15	3298.00	3142.18	3326.00
3328.61	3240.55	3264.34	3306.77
3331.27	3083.75	3212.02	3297.33
3165.62	3202.06	3229.56	3256.65
3379.13	3211.71	3216.80	3174.37
3406.40	3283.97	3236.10	3262.44
3410.19	3171.96	3287.37	3179.22
3317.31	3159.12	3212.34	3274.37
3291.33	3309.41	3438.35	3284.90
3266.48	3281.91	3237.94	3158.09
3267.67	3323.35	3301.72	3101.34
3376.28	3143.22	3290.95	3153.93
3397.17	3216.96	3310.11	3173.91
3172.76	3164.75	3105.71	3195.21
3126.53	3155.45	3415.74	3316.28
3300.40	3348.64	3399.53	3205.99
3201.23	3209.45	3292.04	3365.35
3252.64	3147.86	3339.21	3289.92
3349.13	3212.03	3310.17	3261.92
3040.25	3197.37	3179.35	3125.94
3158.13	3176.30	3265.68	3226.87
3155.96	3149.50	3330.09	3135.10

3225.78	3092.61	3318.22	3280.97
3261.26	3268.72	3353.00	3256.15
3162.47	3360.46	3240.79	3350.05
3257.69	3273.25	3315.63	3176.55
3100.19	3295.22	3365.64	3188.91
3235.27	3264.71	3191.29	3136.53
3281.55	3174.68	3429.76	3277.02
3318.43	3273.54	3327.08	3246.55
3304.52	3151.17	3402.68	3234.43
3363.11	3326.30	3216.58	3248.92
3151.66	3371.09	3327.97	3291.54
3275.36	3253.73	3273.71	3279.86
3198.51	3308.06	3096.40	3087.41
3310.21	3278.49	3278.98	3172.28
3302.72	3286.61	3400.90	3262.26
3299.93	3312.47	3131.85	3173.88
3211.06	3164.41	3174.11	3293.24
3254.84	3205.64	3366.37	3344.07
3274.90	3223.11	3253.52	3249.93
3425.57	3350.50	3188.13	3240.24
3323.09	3213.18	3285.95	3321.59
3452.63	3251.58	3235.56	3180.87
3415.02	3265.69	3226.87	3336.15
3321.91	3156.84	3295.06	3206.52
3297.32	3130.69	3303.74	3267.49
3371.52	3237.78	3315.35	3315.90
3197.21	3223.40	3273.91	3197.99
3386.34	3260.05	3291.10	3340.30
3384.87	3389.81	3238.59	3320.39
3427.13	3085.61	3153.28	3361.94
3286.12	3064.28	3296.37	3285.42
3368.75	3339.78	3341.73	3175.12
3229.04	3266.01	3219.24	3136.60
3340.33	3219.65	3234.17	3301.59
3313.93	3378.35	3364.89	3267.13
3329.94	3256.52	3317.63	3307.63
3347.50	3195.56	3221.05	3239.64
3416.80	3343.22	3292.56	3191.55
3316.72	3166.20	3372.44	3321.32
3384.02	3256.54	3229.18	3271.18
3202.13	3226.49	3299.69	3319.42
3287.28	3312.64	3286.91	3057.34
3249.01	3198.11	3243.69	3307.39
3171.70	3226.64	3192.84	3241.93
3359.29	3086.27	3366.56	3268.58
3217.04	3150.07	3436.68	3218.62
3363.84	3280.01	3251.08	3392.77

3181.99	3160.64	3299.74	3326.36
3274.76	3305.11	3302.87	3050.03
3296.03	3163.81	3277.90	3270.44
3344.93	3281.34	3149.51	3170.89
3338.65	3267.83	3231.26	3221.81
3281.76	3250.13	3286.98	3235.85
3337.90	3300.37	3371.41	3336.94
3403.42	3227.19	3336.35	3326.60
3132.56	3137.95	3250.13	3143.87
3221.02	3180.15	3251.13	3354.80
3166.55	3236.86	3311.82	3225.47
3319.30	3259.47	3311.71	3246.88
3297.26	3343.87	3259.28	3128.38
3318.36	3396.35	3275.62	3235.89
3286.77	3308.00	3188.64	3267.91
3223.30	3334.44	3290.37	3292.77
3242.50	3406.22	3263.27	3359.35
3296.10	3253.99	3319.99	3371.34
3314.69	3336.70	3318.55	3263.65
3144.98	3194.81	3304.43	3072.34
3286.23	3136.55	3351.80	3261.80
3155.12	3165.25	3266.64	3094.65
3245.01	3324.94	3252.46	3280.85
3294.93	3265.17	3368.77	3291.01
3232.40	3302.86	3272.40	3359.58
3301.09	3196.39	3304.26	3328.85
3241.20	3143.11	3306.62	3387.91
3225.28	3116.04	3239.25	3351.52
3287.77	3298.50	3190.38	3185.35
3165.22	3329.98	3060.18	3322.38
3333.61	3205.88	3308.99	3301.36
3252.07	3348.67	3235.63	3241.39
3265.41	3342.91	3322.27	3209.09
3143.02	3248.31	3332.24	3218.79
3285.96	3362.99	3304.53	3265.52
3213.56	3304.21	3279.54	3277.48
3381.24	3229.91	3125.96	3178.44
3310.54	3128.51	3210.78	3217.70
3082.82	3269.37	3275.44	3403.85
3396.69	3151.78	3261.63	3224.83
3243.65	3246.49	3247.69	3187.98
3347.85	3331.85	3401.23	3419.50
3323.78	3150.57	3291.95	3301.04
3431.51	3308.00	3335.66	3323.09
3345.12	3260.38	3267.06	3150.90
3259.42	3307.21	3200.13	3343.51
3234.42	3313.44	3318.95	3455.44

3191.58	3191.44	3212.52	3341.74
3269.50	3398.26	3309.13	3237.01
3213.29	3227.87	3213.36	3165.38
3271.89	3191.90	3314.14	3357.09
3307.09	3156.68	3300.57	3151.08
3367.56	3125.35	3351.14	3318.58
3277.13	3253.19	3253.47	3220.57
3338.24	3262.26	3302.13	3161.73
3234.70	3305.14	3134.73	3367.28
3368.22	3187.68	3274.77	3188.40
3366.56	3222.05	3202.14	3318.53
3275.87	3187.79	3252.08	3360.56
3378.83	3157.91	3212.73	3383.71
3356.08	3206.29	3368.21	3266.70
3300.20	3303.22	3394.08	3236.54
3266.89	3281.08	3340.48	3319.38
3352.91	3122.03	3321.59	3211.64
3358.05	3188.28	3192.09	3168.47
3219.18	3299.95	3219.05	3225.94
3167.82	3326.64	3400.16	3262.96
3278.73	3186.55	3166.95	3370.59
3274.16	3328.09	3282.13	3180.00
3277.78	3278.18	3415.09	3160.72
3328.21	3357.39	3340.05	3153.19
3231.69	3226.58	3322.59	3097.74
3175.37	3286.50	3271.93	3237.91
3383.61	3219.56	3351.00	3349.56
3348.75	3194.34	3311.39	3201.98
3388.26	3184.94	3308.18	3273.30
3309.36	3128.11	3336.25	3245.88
3413.82	3390.94	3195.35	3400.70
3273.83	3375.65	3216.71	3299.38
3417.39	3225.66	3285.60	3352.42
3295.34	3272.09	3346.47	3319.18
3331.36	3204.74	3385.88	3330.66
3213.59	3179.41	3432.95	3197.64
3254.72	3319.61	3198.63	3083.93
3276.91	3401.44	3189.02	3233.33
3309.40	3305.77	3345.46	3082.38
3267.81	3243.34	3307.90	3361.54
3236.44	3329.89	3454.64	3215.63
3368.07	3179.26	3324.56	3258.72
3182.49	3376.92	3222.00	3322.48
3211.91	3323.41	3153.46	3248.98
3308.03	3214.09	3322.71	3288.49
3291.74	3285.72	3274.99	3218.39
3145.35	3256.57	3323.68	3317.38

3325.21	3294.71	3331.98	3188.34
3312.08	3225.18	3302.45	3291.98
3158.61	3294.21	3272.07	3413.05
3396.01	3193.19	3274.15	3320.07
3242.00	3183.12	3298.62	3350.62
3262.69	3280.60	3421.58	3239.28
3444.35	3195.50	3231.55	3328.08
3219.37	3252.70	3175.53	3284.78
3329.38	3115.02	3289.12	3422.64
3370.22	3335.57	3346.64	3097.52
3277.13	3232.67	3212.40	3331.49
3341.59	3306.05	3247.66	3242.15
3334.00	3205.32	3345.97	3248.05
3316.33	3139.07	3287.51	3197.10
3246.84	3152.41	3353.53	3424.39
3214.36	3241.42	3296.87	3303.09
3308.43	3304.24	3241.72	3378.15
3213.10	3168.20	3202.17	3414.03
3233.20	3169.05	3225.46	3050.45
3261.90	3355.05	3238.10	3297.29
3166.93	3211.64	3238.32	3238.73
3289.39	3162.96	3208.01	3240.31
3283.11	3246.74	3157.41	3021.45
3280.51	3360.06	3285.87	3199.45
3048.96	3265.54	3149.36	3200.63
3263.79	3191.60	3117.07	3270.37
3306.37	3297.72	3250.28	3200.77
3232.50	3285.45	3277.50	3285.19
3175.88	3321.44	3174.00	3270.20
3186.58	3257.81	3187.63	3145.05
3257.93	3249.60	3300.11	3268.01
3365.98	3212.46	3232.89	3182.85
3293.97	3343.38	3287.72	3193.05
3290.52	3200.74	3227.01	3255.40
3411.41	3124.20	3301.09	3275.87
3219.89	3320.85	3319.62	3308.05
3355.82	3280.81	3186.87	3292.89
3455.76	3316.60	3219.18	3270.28
3159.35	3267.58	3325.66	3097.26
3184.64	3186.75	3334.74	3246.22
3264.47	3284.22	3293.21	3301.79
3192.34	3156.50	3223.18	3169.27
3181.73	3345.37	3371.09	3179.48
3201.57	3396.83	3308.41	3308.44
3372.68	3261.09	3083.52	3178.57
3287.49	3356.65	3301.95	3303.80
3244.66	3258.30	3142.79	3285.27

3298.10	3316.95	3258.64	3297.05
3301.80	3196.93	3200.86	3215.67
3337.85	3247.97	3223.15	3225.70
3131.76	3388.96	3172.29	3281.19
3347.37	3288.47	3363.10	3329.80
3343.55	3118.52	3180.62	3227.02
3266.85	3222.37	3265.14	3252.74
3492.01	3183.52	3206.56	3222.53
3387.10	3244.14	3258.44	3349.85
3381.36	3296.81	3219.76	3219.38
3341.79	3152.39	3353.92	3115.65
3329.02	3335.38	3274.61	3220.96
3275.30	3197.63	3383.34	3298.00
3382.63	3120.69	3433.71	3283.02
3209.68	3220.80	3367.44	3429.68
3409.16	3373.25	3394.71	3383.76
3108.91	3369.69	3214.75	3184.12
3358.77	3257.93	3202.50	3427.89
3321.97	3231.97	3367.34	3230.98
3320.65	3332.87	3307.81	3265.25
3264.68	3307.26	3251.65	3161.26
3126.11	3142.64	3161.77	3400.62
3297.24	3336.16	3202.74	3349.61
3227.36	3098.00	3129.46	3208.04
3349.73	3321.86	3245.16	3332.07
3116.99	3377.10	3318.25	3324.33
3343.37	3184.48	3272.32	3353.45
3195.86	3310.99	3263.38	3151.65
3430.90	3211.20	3380.08	3221.93
3221.39	3191.01	3322.06	3221.31
3332.75	3239.36	3246.74	3345.58
3385.73	3168.41	3314.45	3217.61
3306.84	3113.89	3397.83	3152.62
3234.78	3133.82	3440.55	3271.20
3212.94	3128.75	3479.52	3287.85
3237.36	3232.34	3296.91	3366.25
3333.76	3339.77	3395.21	3354.64
3262.04	3289.32	3311.84	3199.08
3276.84	3371.83	3373.78	3213.53
3318.57	3216.86	3279.42	3225.04
3270.60	3294.79	3330.33	3074.04
3425.92	3282.57	3316.89	3170.86
3323.83	3271.21	3233.48	3029.62
3352.89	3134.08	3275.59	3185.39
3165.41	3290.42	3184.73	3177.41
3316.41	3270.49	3161.89	3277.28
3283.24	3175.15	3293.98	3314.56

3280.29	3213.29	3337.53	3327.00
3410.53	3385.25	3299.35	3345.06
3273.01	3300.48	3301.52	3221.13
3260.74	3205.60	3304.84	3328.55
3223.19	3179.58	3316.65	3215.44
3228.91	3255.47	3268.33	3073.32
3319.03	3250.32	3151.21	3326.55
3267.61	3380.48	3246.46	3272.40
3263.21	3221.53	3231.62	3247.81
3361.33	3310.62	3297.66	3215.74
3316.61	3395.45	3299.93	3309.67
3380.12	3340.10	3146.74	3331.39
3328.82	3084.85	3310.69	3336.83
3208.71	3298.40	3329.68	3251.32
3385.75	3228.77	3364.28	3284.60
3084.54	3280.58	3438.78	3386.49
3373.46	3283.27	3422.41	3452.35
3251.23	3235.40	3286.74	3329.44
3320.89	3227.24	3215.92	3238.69
3292.31	3247.15	3307.51	3238.14
3484.47	3228.50	3336.50	3402.64
3376.70	3174.20	3269.24	3241.53
3345.34	3350.03	3234.36	3227.07
3280.68	3343.32	3254.58	3331.70
3310.38	3301.14	3366.97	3392.61
3353.00	3242.45	3432.10	3214.97
3281.21	3347.88	3308.07	3260.26
3315.88	3155.46	3325.23	3294.68
3248.37	3249.10	3360.45	3269.77
3280.97	3234.92	3332.07	3323.20
3382.44	3294.46	3254.30	3283.02
3230.07	3122.35	3299.64	3167.32
3202.43	3300.55	3287.19	3361.96
3367.99	3325.86	3236.37	3115.60
3340.73	3319.99	3284.07	3264.29
3327.45	3246.74	3073.42	3282.73
3303.04	3092.75	3199.09	3307.91
3413.88	3304.18	3262.24	3247.47
3236.34	3243.47	3281.39	3249.85
3412.91	3174.97	3233.46	3227.32
3211.59	3258.24	3423.88	3334.19
3204.30	3309.57	3257.46	3449.55
3278.02	3188.23	3306.47	3390.31
3277.78	3184.71	3249.07	3249.85
3291.99	3166.55	3187.32	3357.05
3224.13	3258.69	3292.73	3249.81
3245.29	3317.46	3324.80	3168.60

3307.63	3231.56	3330.11	3247.35
3172.48	3242.52	3149.27	3247.64
3379.90	3293.38	3037.19	3396.66
3377.84	3237.92	3216.63	3267.72
3138.13	3253.40	3372.64	3241.84
3353.95	3176.74	3180.65	3143.24
3343.60	3278.75	3290.37	3307.18
3224.64	3378.98	3175.88	3435.68
3196.47	3341.69	3279.20	3312.18
3211.13	3146.13	3151.17	3211.54
3290.36	3299.43	3223.44	3204.85
3206.55	3284.30	3351.74	3316.49
3219.62	3208.26	3271.01	3316.19
3277.16	3298.31	3302.44	3181.52
3218.60	3330.38	3362.82	3237.11
3186.18	3261.95	3186.83	3120.60
3250.12	3220.74	3241.07	3216.34
3356.73	3238.80	3239.82	3246.62
3223.48	3259.70	3299.13	3379.03
3333.73	3306.44	3264.46	3387.41
3390.12	3221.44	3237.36	3376.46
3306.20	3187.39	3357.16	3279.74
3422.25	3092.23	3179.93	3309.69
3234.38	3133.06	3301.83	3201.88
3377.00	3215.19	3362.91	3274.13
3423.65	3186.07	3260.34	3232.86
3307.64	3069.44	3353.24	3151.59
3165.54	3291.87	3190.25	3262.93
3344.20	3421.35	3176.13	3287.62
3115.83	3136.28	3381.72	3186.92
3223.77	3304.05	3349.72	3268.02
3306.11	3307.56	3245.38	3308.33
3251.98	3324.39	3203.91	3199.85
3366.76	3230.92	3329.33	3377.51
3308.28	3300.50	3316.51	3181.69
3271.55	3128.13	3325.16	3235.00
3237.10	3218.08	3378.90	3230.49
3133.00	3268.94	3388.28	3331.22
3331.46	3281.08	3322.39	3337.18
3401.21	3069.27	3437.67	3380.99
3285.38	3142.23	3374.30	3306.97
3269.14	3283.38	3107.70	3367.51
3223.63	3192.05	3225.54	3225.10
3377.44	3210.10	3329.41	3271.61
3427.19	3152.00	3334.44	3294.16
3289.95	3251.03	3218.14	3251.27
3247.61	3198.87	3184.66	3015.63

3411.84	3427.66	3346.00	3288.89
3386.15	3371.73	3337.14	3416.47
3333.76	3360.71	3274.28	3271.55
3314.54	3264.30	3263.74	3415.70
3323.71	3246.98	3472.81	3394.73
3210.34	3318.21	3301.51	3347.45
3284.11	3343.53	3194.58	3230.66
3315.19	3240.54	3094.37	3341.21
3311.29	3306.67	3308.73	3092.96
3266.27	3213.97	3255.14	3328.82
3198.46	3393.58	3375.09	3333.28
3296.62	3311.71	3186.24	3135.97
3200.13	3289.46	3266.68	3317.77
3471.22	3161.55	3360.78	3243.48
3251.37	3228.21	3320.28	3185.32
3378.23	3179.59	3293.12	3256.72
3383.71	3273.35	3311.50	3294.93
3281.53	3246.28	3289.02	3285.51
3321.88	3300.60	3235.65	3311.67
3373.18	3190.74	3318.22	3404.20
3287.89	3265.56	3224.86	3381.84
3412.81	3284.38	3332.07	3268.64
3358.10	3198.17	3171.23	3246.34
3332.49	3285.06	3306.25	3222.92
3280.41	3259.89	3420.62	3207.19
3409.21	3207.21	3122.41	3192.96
3301.73	3216.68	3146.17	3355.56
3288.30	3305.21	3257.03	3292.58
3168.00	3211.32	3293.39	3131.21
3225.02	3184.66	3185.10	3363.97
3307.90	3155.51	3331.47	3341.46
3288.24	3300.21	3365.57	3360.21
3267.03	3300.40	3326.64	3311.85
3357.39	3183.01	3245.07	3388.40
3170.83	3245.05	3164.07	3250.06
3354.62	3330.65	3244.69	3255.10
3189.08	3073.17	3357.12	3319.34
3261.23	3243.80	3385.32	3133.35
3234.34	3211.33	3188.70	3320.00
3262.79	3256.43	3366.33	3360.38
3292.72	3256.45	3380.99	3375.47
3315.83	3219.45	3086.52	3289.22
3167.70	3328.43	3067.18	3187.87
3348.54	3242.26	3262.02	3286.91
3053.47	3199.38	3135.56	3325.76
3218.47	3237.63	3279.45	3237.33
3194.85	3194.14	3311.89	3356.89

3329.69	3168.77	3308.66	3155.14
3285.28	3161.01	3340.34	3256.22
3115.42	3230.52	3182.93	3245.25
3219.84	3243.93	3366.86	3294.86
3194.10	3036.50	3234.47	3327.70
3358.89	3360.18	3207.65	3304.74
3381.22	3379.91	3353.83	3352.65
3384.23	3342.23	3288.69	3331.88
3369.24	3283.76	3264.62	3316.08
3259.31	3256.30	3339.57	3246.12
3327.25	3302.00	3291.36	3303.09
3105.95	3201.22	3313.49	3066.43
3205.38	3277.07	3350.49	3181.27
3300.22	3378.76	3398.89	3183.54
3306.94	3210.73	3279.55	3187.37
3209.53	3168.25	3292.15	3352.65
3063.19	3135.64	3304.77	3279.88
3299.34	3266.58	3136.49	3323.59
3324.71	3167.74	3285.41	3299.72
3224.26	3274.38	3192.50	3304.23
3300.37	3280.11	3162.17	3174.49
3398.42	3235.53	3238.43	3090.35
3323.07	3377.98	3274.70	3422.87
3228.59	3346.23	3345.44	3061.62
3347.74	3318.18	3382.38	3374.45
3310.60	3253.79	3325.64	3255.26
3301.33	3171.84	3409.46	3235.72
3358.41	3090.58	3365.52	3358.58
3344.83	3221.20	3187.02	3088.62
3316.77	3386.25	3199.62	3357.62
3335.15	3331.07	3177.47	3319.02
3362.92	3251.19	3072.27	3252.80
3216.82	3417.46	3358.42	3344.89
3335.34	3181.37	3250.66	3202.83
3205.73	3413.23	3320.49	3233.60
3324.89	3204.26	3289.15	3388.25
3381.00	3261.05	3171.81	3357.54
3247.69	3177.98	3193.82	3348.92
3390.12	3291.98	3389.75	3304.12
3262.70	3291.89	3332.99	3380.87
3161.08	3228.85	3335.35	3139.71
3252.86	3295.66	3320.39	3322.65
3117.04	3200.09	3254.46	3385.44
3258.70	3264.58	3192.10	3210.75
3165.87	3244.01	3214.21	3171.12
3401.59	3247.07	3273.31	3179.48
3283.15	3252.64	3303.97	3380.52

3160.73	3241.95	3331.61	3219.98
3359.55	3362.89	3381.31	3408.58
3259.94	3217.44	3264.69	3363.21
3253.46	3257.37	3219.62	3256.13
3295.87	3242.23	3204.74	3354.34
3225.93	3226.59	3255.52	3426.15
3253.37	3236.39	3088.30	3339.63
3407.24	3187.75	3056.95	3272.82
3328.72	3343.37	3233.47	3407.05
3380.84	3279.04	3282.24	3340.84
3181.12	3330.23	3273.91	3291.33
3321.57	3199.83	3419.19	3238.37
3233.90	3121.35	3317.24	3334.85
3292.82	3304.66	3343.64	3229.03
3288.77	3173.89	3335.72	3325.13
3082.91	3348.45	3338.71	3257.65
3129.95	3160.57	3125.16	3316.48
3337.79	3191.28	3278.49	3311.17
3390.09	3177.35	3264.53	3389.56
3290.37	3352.87	3315.01	3334.24
3220.23	3208.11	3287.33	3407.00
3179.37	3405.39	3406.70	3397.69
3208.28	3304.50	3405.73	3211.96
3333.22	3368.14	3137.92	3253.40
3334.91	3313.72	3251.77	3296.03
3250.98	3345.55	3364.36	3226.92
3471.18	3260.32	3210.19	3255.06
3192.61	3257.65	3320.50	3098.43
3374.16	3264.23	3409.09	3383.77
3331.89	3209.09	3180.13	3222.49
3141.98	3248.78	3317.32	3161.52
2897.70	3403.45	3310.51	3262.57
2482.05	3216.88	3264.79	3293.13
2310.26	3321.38	3353.33	3264.51
2199.85	3218.80	3397.94	3248.13
2128.50	3293.51	3258.08	3273.62
2067.66	3227.02	3223.72	3360.35
2019.40	3150.41	2733.06	3303.91
1980.57	3348.95	2443.20	3291.00
1946.97	3261.12	2315.71	3328.64
1918.49	3200.77	2221.48	3340.50
1892.19	3304.46	2153.26	3171.96
1868.14	3102.44	2098.28	3395.83
1848.80	3237.33	2053.86	3322.88
1831.91	3241.47	2016.78	3231.51
1812.68	3267.79	1980.35	3308.34
1799.75	3147.71	1951.21	3331.86

1785.48	2664.49	1923.36	3363.28
	2388.92	1900.03	3327.36
	2273.35	1878.66	3408.96
	2189.38	1858.63	3231.34
	2124.09	1840.41	3250.18
	2073.79	1825.19	3359.30
	2033.28	1812.22	3177.88
	1996.15	1799.09	3253.24
	1965.74	1785.66	3148.04
	1937.68	1774.04	3252.06
	1916.18	1763.31	3373.77
	1894.33	1751.52	3318.05
	1875.18	1741.41	3199.90
	1856.55	1730.51	3108.65
	1840.43	1720.26	3327.33
	1823.84	1712.25	3363.42
	1808.43	1703.45	3290.90
	1797.10	1695.59	3331.95
	1783.23	1690.01	3220.16
	1770.82	1680.95	3037.67
	1759.53	1676.73	3216.78
	1747.80	1670.91	3391.01
	1740.94	1667.12	3192.38
	1729.16	1662.40	3319.04
	1721.43	1653.47	3154.73
	1711.66	1648.50	3297.60
	1702.70	1642.41	3359.93
	1696.57	1637.31	3353.47
	1689.71	1634.12	3200.45
	1680.54	1628.21	3271.44
	1674.77	1624.28	3260.89
	1668.45	1618.69	3316.95
	1663.18	1617.13	3096.03
	1659.36	1609.56	3065.72
	1651.33	1609.16	3278.27
	1650.14	1604.34	3317.27
	1641.76	1598.47	3197.06
	1637.89	1599.61	3237.52
	1631.97	1595.09	3382.79
	1627.68	1587.85	3343.61
	1625.00	1587.07	3304.72
	1621.70	1580.46	3318.96
	1615.24	1578.93	3166.74
	1609.83	1578.95	3393.89
	1606.82	1574.20	3265.10
	1604.49	1569.51	3305.32
	1599.90	1568.73	3360.06

	1593.90	1555.21	3297.56
	1593.63	1559.62	3286.29
	1587.99	1557.54	3303.02
	1585.16	1551.75	3298.83
	1581.38	1554.35	3283.10
	1577.49	1550.43	3218.37
	1574.79	1546.47	3267.47
	1570.02	1547.85	3207.58
	1566.35	1539.37	3337.96
	1564.05	1537.21	3365.68
	1563.99	1530.70	3271.33
	1560.98	1535.73	3352.04
	1555.13	1531.41	3278.71
	1556.62	1530.83	3165.72
	1548.92	1524.50	3257.60
	1547.73	1527.68	3268.92
	1545.10	1520.06	3222.85
	1546.66	1530.57	3296.14
	1539.99	1519.86	3273.97
	1536.91	1513.65	3372.57
	1536.50	1508.92	3216.42
	1537.52	1510.11	3299.93
	1532.03	1514.44	3334.84
	1530.57	1510.96	3295.33
	1526.27	1509.06	3344.08
	1528.01	1508.52	3271.41
	1522.31	1502.41	3134.45
	1521.46	1508.24	3225.16
	1524.94	1503.55	3225.35
	1516.17	1502.81	3294.95
	1518.96	1500.49	3324.34
	1510.57	1495.55	3371.69
	1513.39	1496.66	3245.26
	1516.45	1501.76	3220.24
	1506.43	1493.81	3133.87
	1508.51	1494.78	3212.08
	1506.96	1489.71	3193.45
	1505.95	1490.69	3350.54
	1502.41	1490.87	3418.71
	1501.85	1493.91	3346.25
	1499.38	1482.12	3364.36
	1497.29	1483.87	3230.53
	1496.97	1493.57	3160.99
	1495.83	1484.09	3387.20
	1492.56	1488.38	3320.93
	1498.04	1478.55	3346.41
	1496.17	1484.95	3333.47

	1488.65	1483.14	3318.32
	1485.12	1479.61	3120.06
	1485.60	1479.99	3223.66
	1490.67	1479.97	3435.22
	1481.69	1470.73	3310.87
	1486.43	1478.77	3310.57
	1483.25	1475.27	3320.50
	1474.41	1467.49	3200.62
	1480.24	1468.71	3315.07
	1477.61	1467.61	3302.17
	1479.40	1471.64	3197.83
	1486.83	1470.24	3306.87
	1480.82	1469.32	3374.03
	1480.19	1469.26	3391.06
	1483.48	1468.10	3261.03
	1475.90	1462.99	3233.15
	1479.14	1462.11	3127.58
	1470.02	1461.12	3418.32
	1478.25	1465.69	3272.54
	1478.96	1461.44	3126.70
	1481.08	1454.53	3122.89
	1469.04	1457.50	3253.88
	1465.49	1454.66	3324.06
	1470.97	1461.67	3315.84
	1470.60		3352.72
	1473.93		3398.35
	1471.77		3326.99
	1470.67		3393.57
	1461.14		3272.48
	1470.08		3249.18
	1468.02		3244.88
	1463.90		3137.83
	1467.50		3202.46
	1466.45		3125.31
	1462.77		3114.14
	1464.32		3248.58
	1465.74		3376.41
	1460.91		3286.47
	1462.10		3304.35
	1466.24		3297.50
	1465.02		3315.10
	1461.43		3229.97
	1458.59		3421.80
	1456.15		3301.00
	1459.21		3262.75
	1453.98		3311.27
	1460.33		3175.54

	1462.05		3261.66
	1463.44		3233.34
	1463.63		3332.67
	1458.56		3187.78
	1455.24		3379.77
	1458.92		3376.46
	1450.84		3276.63
	1470.26		3337.59
	1466.16		3259.71
	1463.64		3255.82
	1451.50		3194.67
	1461.64		3230.32
	1459.02		3138.54
	1451.54		3282.49
	1455.00		3325.77
	1459.75		3198.07
	1455.45		3157.41
	1458.59		3391.93
	1460.65		3230.16
	1452.13		3443.54
	1460.66		3199.24
	1459.46		3320.50
	1456.82		3375.91
	1454.36		3392.84
	1447.78		3335.53
	1457.24		3330.74
	1456.95		3420.38
	1448.64		3323.91
	1455.28		3385.02
	1453.68		3328.38
	1450.19		3387.79
	1457.07		3375.79
	1446.13		3266.62
	1446.51		3290.14
	1452.15		3370.82
	1450.82		3280.95
	1460.20		3246.39
	1455.67		3258.80
	1451.79		3236.97
	1452.42		3401.63
	1453.09		3228.45
	1453.27		3112.25
	1449.29		3395.77
	1451.21		3280.02
	1454.86		3378.89
	1455.61		3200.57
			3340.41

			3253.30
			3299.79
			3167.91
			3367.30
			3359.60
			3383.64
			3277.44
			3318.73
			3233.53
			3217.36
			3273.96
			3249.45
			3196.58
			3134.70
			3261.60
			3295.71
			3165.21
			3364.74
			3283.85
			3061.67
			3378.30
			3213.51
			3331.68
			3110.15
			3390.71
			3193.46
			3265.30
			3307.14
			3359.75
			3215.95
			3190.59
			3448.43
			3392.93
			3345.72
			3257.69
			3245.61
			3324.86
			3255.94
			3294.15
			3118.15
			3409.75
			3388.33
			3281.73
			3131.47
			3352.56
			3265.31
			3215.66

			3292.64
			3355.86
			3462.11
			3113.61
			3106.55
			3420.43
			3280.50
			3365.17
			3207.55
			3288.97
			3334.47
			3213.68
			3275.25
			3217.69
			3279.44
			3242.97
			3484.09
			3373.88
			3402.85
			3356.33
			3346.62
			3288.79
			3257.31
			3310.73
			3379.62
			3300.79
			3184.55
			3217.86
			3219.57
			3311.83
			3282.38
			3240.41
			3209.02
			3257.10
			3269.06
			3192.32
			3406.48
			3333.38
			3307.74
			3217.48
			3245.57
			3305.79
			3315.01
			3230.53
			3247.86
			3275.19
			3256.55

			3353.77
			3331.12
			3217.58
			3314.94
			3116.86
			3318.49
			3115.34
			3177.27
			3330.49
			3223.34
			3291.23
			3380.13
			3360.30
			3358.52
			3429.61
			3124.68
			3302.54
			3189.50
			3259.49
			3169.91
			3216.78
			3327.33
			3354.29
			3355.97
			3224.10
			3341.96
			3462.73
			3228.36
			3340.28
			3301.90
			3344.53
			3183.66
			3219.36
			3134.20
			3133.09
			3255.78
			3491.06
			3343.02
			3385.70
			3359.39
			3340.81
			3194.07
			3312.78
			3368.28
			3137.22
			2672.64
			2437.56

			2342.21
			2255.72
			2187.28
			2133.82
			2090.94
			2051.73
			2015.20
			1986.45
			1960.22
			1937.26
			1914.27
			1896.32
			1880.11
			1863.10
			1849.00
			1835.62
			1824.19
			1808.25
			1797.41
			1786.15
			1776.11
			1766.94
			1755.25
			1744.05
			1734.42
			1726.13
			1716.96
			1706.91
			1698.48
			1695.08
			1687.61
			1681.02
			1673.61
			1669.00
			1663.88
			1659.18
			1653.06
			1648.43
			1643.33
			1638.05
			1632.45
			1631.17
			1624.55
			1621.43
			1616.45
			1614.87
			1610.07

			1606.95
			1604.19
			1601.41
			1596.76
			1592.46
			1588.88
			1582.50
			1582.72
			1576.46
			1571.25
			1571.60
			1575.48
			1565.42
			1566.82
			1563.70
			1557.31
			1558.18
			1552.62
			1557.36
			1549.31
			1545.74
			1545.06
			1537.13
			1539.53
			1536.97
			1531.26
			1535.13
			1531.20
			1527.08
			1528.19
			1526.73
			1519.36
			1521.29
			1519.10
			1521.83
			1517.29
			1520.35
			1509.07
			1512.76
			1515.71
			1503.25
			1502.25
			1505.05
			1506.86
			1501.77
			1503.16
			1502.90

			1499.00
			1495.40
			1493.62
			1493.90
			1493.37
			1498.85
			1492.22
			1496.33
			1488.74
			1498.29
			1488.31
			1477.57
			1479.11
			1478.38
			1481.26
			1487.33
			1479.83
			1481.49
			1479.22
			1473.36
			1480.54
			1479.99
			1471.56
			1478.67
			1472.37
			1472.45
			1470.12

Table S2. Recession data acquired for HARLEM samples.

HARLEM 1		HARLEM 2		HARLEM 3		HARLEM 4	
Time (s)	Recession (μm)	Time (s)	Recession (μm)	Time (s)	Recession (μm)	Time (s)	Recession (μm)
0.00	0.00	0.00	0.00	0.00	0.00	0.00	0.00
0.20	-20.69	0.19	0.13	0.18	-3.48	3.42	26.00
0.40	-51.24	3.32	23.52	0.38	0.00	3.61	37.46
3.82	68.85	3.49	75.20	0.58	-2.53	3.80	46.90
4.00	90.40	3.70	65.41	3.00	194.50	4.00	-34.74
4.20	59.43	3.89	49.98	3.18	201.96	6.42	209.08
4.40	33.14	6.91	235.01	3.38	186.38	6.61	197.98
7.82	225.78	7.09	251.63	3.58	131.77	6.80	272.56
8.01	215.91	7.29	221.59	7.00	351.34	7.01	213.74
8.20	218.63	7.49	256.76	7.18	372.98	10.42	400.20
8.41	188.88	9.91	449.89	7.38	420.08	10.60	428.06
10.82	517.63	10.10	443.56	7.58	375.81	10.81	435.29
11.00	412.82	10.29	430.13	11.00	548.76	11.00	399.93
11.20	419.30	10.49	410.53	11.18	552.93	14.42	553.27

11.40	374.98	13.91	582.23	11.38	548.78	14.61	586.66
14.82	598.54	14.09	628.14	11.58	541.69	14.80	553.24
15.00	618.68	14.29	630.06	15.00	737.61	15.00	560.48
15.20	633.52	14.49	606.95	15.18	734.15	17.42	554.94
15.40	603.20	17.91	692.65	15.38	710.08	17.60	573.27
18.82	807.81	18.09	744.28	15.58	675.09	17.80	529.87
19.00	813.91	18.29	719.99	18.00	868.54	18.00	524.06
19.20	799.52	18.49	722.87	18.19	919.03	21.42	630.61
19.40	763.24	21.91	853.37	18.38	911.22	21.60	648.76
21.82	1017.79	22.09	866.27	18.59	907.72	21.80	611.34
22.00	1010.69	22.29	895.39	22.00	1066.99	22.00	584.44
22.20	1015.98	22.49	842.79	22.18	1110.53	25.42	821.88
22.40	992.44	24.91	1111.04	22.38	1057.80	25.61	737.20
25.82	1268.03	25.09	1045.64	22.58	1030.56	25.80	747.46
26.00	1259.13	25.29	1059.39	26.00	1228.31	26.00	680.28
26.21	1253.31	25.49	1065.37	26.18	1238.12	29.42	829.26
26.40	1221.05	28.91	1204.98	26.38	1209.34	29.60	878.62
29.82	1496.00	29.10	1227.95	26.58	1220.78	29.80	899.43
30.00	1499.50	29.29	1229.11			30.00	882.93
30.20	1503.63	29.50	1176.46			32.42	1226.58
30.40	1516.12					32.60	1261.13
						32.80	1224.69
						33.00	1241.62
						36.42	1577.66
						36.60	1584.41
						36.80	1630.45
						37.00	1560.23
						40.42	1925.60
						40.60	1973.76
						40.80	1981.21
						41.00	1913.74
